# Joinpoint analyses of rates on hospital-recorded deliberate self-harm: an update on Danish national trends

**DOI:** 10.1007/s00127-024-02795-y

**Published:** 2024-11-11

**Authors:** Britt Reuter Morthorst, Michella Heinrichsen, Annette Erlangsen

**Affiliations:** 1 Danish Research Institute for Suicide Prevention, Mental Health Centre Copenhagen, Copenhagen, Denmark; 2 Department of Mental Health, Johns Hopkins Bloomberg School of Public Health, Baltimore, MD USA; 3 Copenhagen Research Centre for Mental Health, Copenhagen, Denmark; 4 Center of Mental Health Research, Australian National University, Canberra, Australia; 5 Research Unit, Child and Adolescent Mental Health Center, Copenhagen University Hospital – Mental Health Services CPH, Copenhagen, Denmark; 6 Department of Clinical Medicine, Faculty of Health, University of Copenhagen, Copenhagen, Denmark

**Keywords:** Deliberate self-harm, Cohort study, Joinpoint, Epidemiology, Deliberate self-harm methods.

## Abstract

**Introduction:**

Deliberate self-harm (DSH) is a public health concern and the high rates among adolescents and females warrant continuous monitoring. The aim of this study was to determine trends in DSH rates by gender and age in Denmark during 2000–2021 using joinpoint regression analysis.

**Methods:**

A cohort design was applied to national register data on all individuals aged 10 + years living in Denmark during 2000–2021. DSH episodes were identified in somatic and psychiatric hospital data. Sex- and age-specific incidence rates (IR) were calculated by calendar years. Using joinpoint regression analyses, segments of change and their annual percent change (APC) were identified.

**Results:**

The highest DSH rates were observed for males and females aged 19–24 years with IRs of 146.8 (95% CI 142.9–150.7) and 378.6 (95% CI 372.1–385.0) per 100,000 person-years, respectively. Major changes in DSH rates were found for the youngest age groups. A step decrease was found for males aged 19–24 years (-18.4; 95% CI -31.9- -2.3; p = < 0.030) during 2012 to 2015. A significant decline was observed during 2012–2016 for females aged 19–24 years (-18.9; 95% CI -26.8 – -10.2; *p* = 0.001). Poisoning was the most frequently used method.

**Conclusion:**

Seemingly, the financial recession in 2008 did not affect Danish DSH rates. Significant declines were observed for females in the years where means restrictive measures had been installed; thus, supporting their potential effect. Fluctuations in DSH rates among adolescents and young adults in recent years underscore the importance of continued monitoring.

**Supplementary Information:**

The online version contains supplementary material available at 10.1007/s00127-024-02795-y.

## Introduction

Deliberate self-harm (DSH) is, with an estimated 12-month prevalence of respectively 0.3 and 0.4% in high income and low- and middle-income countries, a public health concern [1]. For this reason, the WHO (World Health Organization) has emphasized the importance of long-term monitoring of DSH incidence rates and methods in order to guide suicide preventive strategies and interventions [2, 3]. The importance of this is further emphasized by the fact that DSH is the one of the most prominent risk factors for death by suicide [4, 5] also among youth [6].

Epidemiological studies based on national hospital records have shown varying but also increasing rates of DSH over recent decades [7, 8]. The financial crisis in 2008 has been suggested as a potential contributing factor for such increases [9], for instance in Ireland where elevated rates of DSH among males were linked to unemployment, financial losses and home repositions [10, 11]. Nevertheless, a 7% increase in DSH rates, which was observed in Ireland between 2013 and 2019, seemingly mainly impacted older adults [12]. In Iceland, the financial crisis was followed by an increase in DSH by paracetamol poisoning; this trend continued to increase despite financial recovery during 2010 to 2017 [13].

Trends of DSH may also have been effectuate through changes in availability of drugs and other methods. For instance, a 40.5% increase in incidence of hospital treated paracetamol poisonings was observed in Sweden in 2013 after the introduction of non-opioid-analgesics in non-pharmacy outlets [14]. On the other hand, restricting access to means has been applied as a suicide preventive strategy in several European countries [15]. In United Kingdom, a pack size restriction, including limited availability of paracetamol, was introduced in 1998 resulted in significant reductions in paracetamol and salicylate overdoses as measured by poisoning admissions between 1993 and 2002 [16]. This effort was also linked to a 43% reduction in paracetamol-overdose deaths in 2009 [17]. A similar pack size restriction on over-the-counter (OTC) analgesics was implemented in Denmark in 2013 and linked to an 18% reduction in hospital contacts for non-opioid-analgesic poisonings as well as reduced severity of poisoning cases in 2015 when compared to 2000 [18]. Also, age restrictive efforts were introduced in Denmark in 2011 implying that only individuals aged 18 years or older were allowed to buy OTC agents [18].

The highest rates of DSH have consistently been reported for adolescents and young adults, especially females [19]. In Ireland, females aged 15-19-years were found to have the highest rate with 620 per 100,000 population, while the highest rate among males was found for those aged 20-24-years with 427 DSH episodes per 100,000 population during 2003 to 2009 [10]. In Norway, the highest rates were found among females aged 15–19 and 20–24 years with rates of 296 and 283 per 100,000 person-years, respectively, during 2008 to 2013 [20]. In 2011 in Denmark, the highest DSH rates were found among females aged 15–19 years who had a rate of 544 per 100,000 person-years, while males aged 20–24 years had a rate of 147 [7]. In Norway, 38% of all hospital recorded DSH episodes occurred among individuals aged 20–34 years, while those aged 65 + years accounted for 5% [20]. DSH is more frequent among females than males [7]. This is also a consistent finding for adolescents and young adults where DSH is about twice as frequent among females when compared to males [21]. Most DSH episodes occur by poisoning [7, 22, 23].

Despite a potential under-recording [1, 24], national administrative registers have been emphasized as a valuable resource for monitoring trends of DSH [25]. The aim of this study was to determine changes in the DSH rates by sex and age groups in Denmark during 2000–2021 using national hospital data and joinpoint analyses.

## Method

A cohort design was applied to national linkage data covering all persons who were living in Denmark at some point between January 1st, 2000, to December 31st, 2021. Individual-level register data was linked using the unique, personal id-number, which is assigned upon birth or first migration into the country. Data from the Civil Registration System was linked with national data on all contacts to somatic and psychiatric hospital from the National Patient Register and the Psychiatric Central Research Registry, respectively. These registers included information on date of admission and discharge, diagnosis, and reason for contact (e.g. suicide attempt or accident) [26, 27]. Hospital data were available for emergency department, inpatient, and outpatient contacts. Diagnoses were recorded according to the 10th revision of WHO International Classification of Diseases (ICD) [28].

### Study population

All individuals aged 10 years or older were observed from January 1st, 2000, date of immigration, or their 10th birthday. The study population was followed until date of emigration, date of death, or December 31st, 2021, whichever occurred first. We opted to include individuals from age 10 years onwards as previous evidence suggest that only few incidents are recorded before this age and these may partly be erroneous records of accidents rather than DSH episodes [29].

### Outcome

DSH episodes were defined as hospital contacts recorded in the National Patient Register or the Psychiatric Research Register where the main or sub-diagnosis indicated a self-harm episode (ICD-10: X60-X84) or where the reason for contact was listed as being suicide attempt (coded as ‘4’ before 2018 and as ‘ALCC04’ thereafter). Individuals could be recorded with several episodes of DHS. During the initial data management, we accounted for transfers between different hospital units to ensure that there were no double-recordings of the same DSH-episode. An individual would first be recorded with a new episode of DSH on the next day after having been discharged from a previous hospital contact due to a DSH-episode, i.e. after approximately 24 h. Individuals who died during a hospital contact for DSH were considered as having died by suicide and, hence, not included.

The operational definition of DSH by the Danish Health Authority aligns with the one by the WHO, i.e. “*an act with a non-fatal outcome*,* in which an individual deliberately initiates a non-habitual behaviour that without intervention from others will cause self-harm*,* or deliberately ingests a substance in excess of the prescribed or generally recognized therapeutic dosage*,* and which is aimed at realizing changes which the subject desired via the actual or expected physical consequences” (page 99)* [30]. As such, the definition refers to non-fatal acts with varying motives, which may include the a wish to die although this is not a prerequisite as seen from the WHO definition presented above [31].

Based on ICD-10 codes, DSH methods were classified as: poisonings (X60-69), hanging (X70), drowning (X71), firearms and explosives (X72-X75), cutting (X78-79), jumping (X80), moving object (X81-X82), and other methods (X76-X77, X83-X84). In addition, additional diagnostic codes, such as poisonings (T36-T65, T96) and lesions on the arm (S50-S69), were screened to identify method used in DSH episodes, which had been identified through the ‘reason for contact’ recording rather than a diagnostic code. If multiple methods were listed; the method was determined from the main diagnosis. Due to relatively few events by other methods and based on preliminary analysis, DSH methods were categorized as poisonings, cutting, and other methods.

### Statistical analyses

Incidence rates (IR) were calculated by dividing number of DSH episodes with the population at risk, which was measured as person-days, and reported per 100,000 person-years with their 95% confidence intervals. IRs were calculated by calendar year (2000, 2001, 2002 …, 2021), sex (males and females), and age group (10–18 years, 19–24 years, 25–44 years, 45–64 years, and 65 + years). Trends in the IRs during 2000 to 2021 were identified using joinpoint analyses. Based on assumptions for Poisson regression models with equally distributed risk, joinpoints, i.e. years where the trend of the IR changed, were identified through permutation tests [32]. Annual percent change (APC) was estimated for each joinpoint segment with 95% confidence intervals. The joinpoint analyses were conducted by sex and age group. We opted to use the above listed age groups as they, in preliminary analyses, were identified to have sufficient events in each calendar year and reflected the age groups, which had been targeted by previous suicide preventive measures in Denmark [18]. Also, changes in methods of DSH were assessed by obtaining the percentual distribution for the years 2000, 2013, 2017, and 2021, which were years identified as joinpoints as well as years of relevance in terms of the implemented pack size restriction on non-opioid analgesics [18].

Data management and analysis was conducted using SAS [33] and the joinpoint Regression Program version 4.9.0.1 [34], respectively.

### Ethical considerations

According to Danish legislation, individual informed consent is not required for register-based studies. Approval from the Danish Protection Agency was obtained (Region H: P-2020-305). Further ethical approval was not needed.

## Results

A total of 2,882,637 males and 2,914,185 females aged 10 years and over were observed over 48,169,133 and 49,227,832 person-years, respectively, during 2000–2021.

In all, 35,646 DSH episodes were recorded for males (median age: 37 years; IQR: 24–49 years) and 63,972 for females (median age: 26 years; IQR: 18–44 years). The overall IRs were 74.0 (95% CI 73.2–74.8) and 130.0 (95% CI 128.9–131.0) per 100,000 person-years for males and females, respectively. Among males, those aged 25–44 years accounted for 41% of all DSH episodes, while those aged 65 years and over accounted for 8.1%. Among females, the corresponding percentages were 29.5% of all episodes observed among those aged 25–44 years and 5.8% among those aged 65 years and over.

### Incidence rates

The highest IRs were observed among young and middle-aged age groups. Males aged 19–24 and 25–44 years had IRs of 146.8 (95% CI 142.9–150.7) and 104.4 (95% CI 102.7–106.1) per 100,000 person-years, respectively (Table 1). The highest absolute IR was observed among females 19–24 years with 378.6 (95% CI 372.1–385.0) per 100,000 person-years and was followed by females aged 10–18 years who had an IR of 263.8 (95% CI 259.8–267.8). The overall male to female rate ratio was 1.8, while the ratio was 2.5 among the 19–24-year-olds was 2.5.

**Table 1:** Incidence rates (IR) of DSH per 100,000 pers on years during 2000–2021

	DSH *n* (%)	Person-years (%)	IR (95% CI)
Males			
Age group			
10–18 years	3564 (10.0)	6,688,021 (13.9)	53.3 (51.5–55.0)
19–24 years	5373 (15.1)	3,660,424 (7.6)	146.8 (142.9–150.7)
25–44 years	14,613 (41.0)	13,995,417 (29.1)	104.4 (102.7–106.1)
45–64 years	9208 (25.8)	14,840,587 (30.8)	62.0 (60.8–63.3)
65 + years	2888 (8.1)	8,984,684 (18.7)	32.1 (31.0–33.3)
Total	35,646 (100)	48,169,133 (100)	74.0 (73.2–74.8)
Females			
Age group			
10–18 years	16,729 (26.2)	6,342,634 (12.9)	263.8 (259.8–267.8)
19–24 years	13,149 (20.6)	3,473,359 (7.1)	378.6 (372.1–385.0)
25–44 years	18,872 (29.5)	13,499,547 (27.4)	139.8 (137.8–141.8)
45–64 years	11,527 (18.0)	14,766,974 (30.0)	78.1 (76.6–79.5)
65 + years	3695 (5.8)	11,145,318 (22.6)	33.2 (32.1–34.2)
Total	63,972 (100)	49,227,832 (100)	130.0 (128.9–131.0)

### Joinpoint segments

**Fig. 1:**
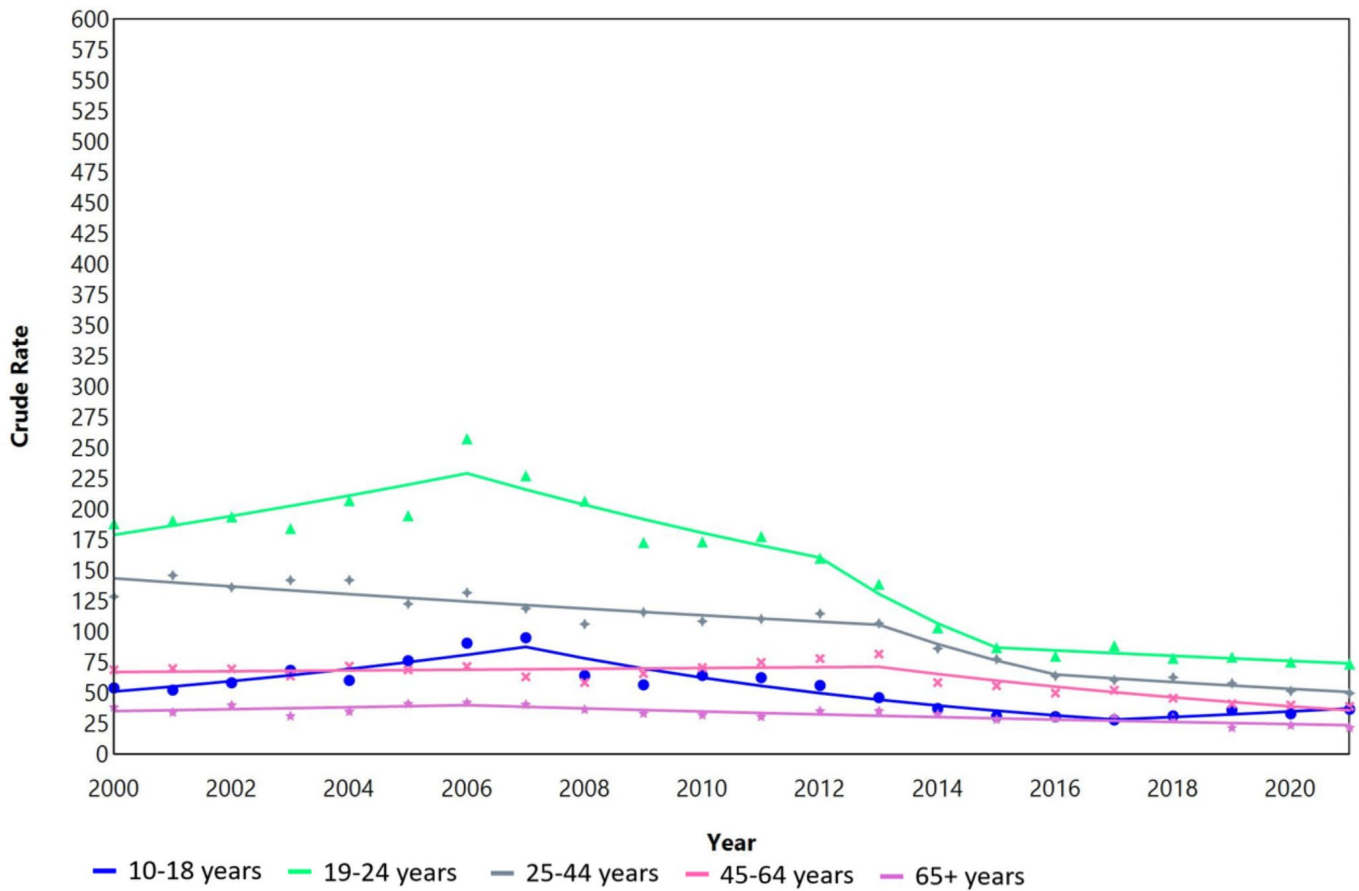
Joinpoint analysis of the incidence rates for DSH per 100,000 person years for males in Denmark during 2000–2021

**Fig. 2:**
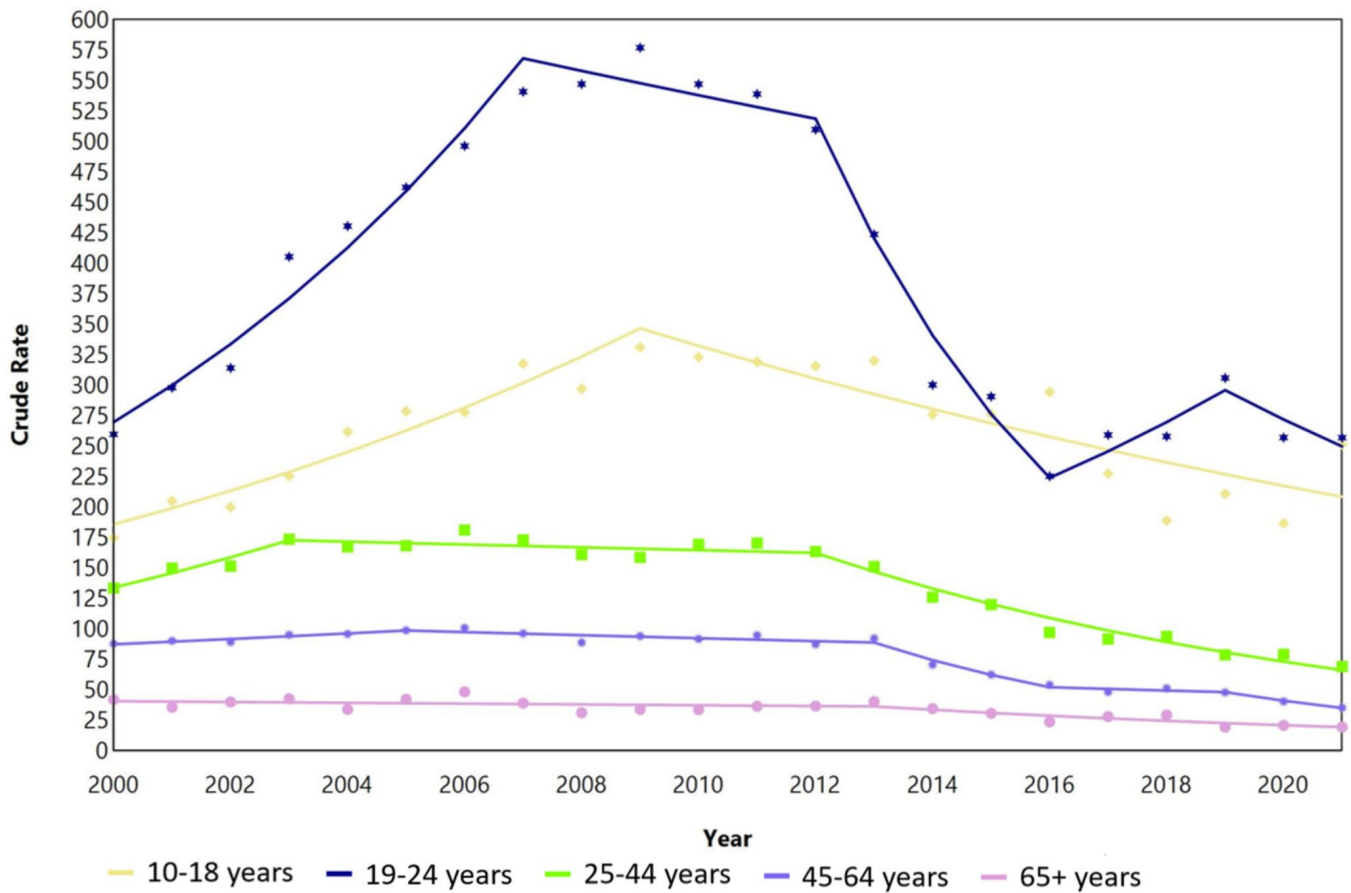
Joinpoint analysis of the incidence rates for DSH per 100,000 person years for females in Denmark during 2000–2021

Between 2 and 5 joinpoint segments were identified by sex and age group (Figs. 1 and 2).

For males aged 10–18 years, an increasing trend with an APC of 8.0 (95% CI, 3.5–12.6; *p* = 0.002) was found during 2000–2007. In the subsequent years, a segment with a decreasing trend was identified during 2007–2017 with an APC of -10.6 (95% CI, -13.6 - -7.6; p = < 0.001) and another segment with an increasing trend during 2017–2021 with an APC of 7.2 (95% CI, -6.2–22.5), although the latter did not reach statistical significance as indicated by the confidence interval. For males aged 19–24 years, significantly decreasing trend segments were observed during 2006–2012 (APC, -5.8; 95% CI, -8.9 - -2.6; p = < 0.001) and 2012–2015 (APC, -18.4; 95% CI, -31.9 - -2.3; *p* < 0.001). Decreasing trends were observed over the entire period, albeit at different paces, for males aged 25–44 years and those aged 65 years and over (Table 2).

Among young females aged 10–18 years, a significant increasing trend (APC, 7.2; 95% CI, 4.2–10.2; *p* < 0.001) was observed during 2000–2009, followed by a significant decrease during 2009–2021 (APC, -4.2; 95% CI, -5.7- -2.5; p = < 0.001). The IR of females aged 19–24 years underwent a significant increase during 2000 till 2007 (APC, 11.2; 95% CI, 8.0-14.5; *p* < 0.001), which was followed by a decreasing segment during 2012–2016 (APC, -18.9; 95% CI -26.8- -10.2; *p* = 0.001). In recent years, significant declines were also observed among females aged 45–64 years (2013–2016: APC, -16.2 (95% CI -26.2 - -4.9; *p* = 0.012) and those aged 65 years and over (2013–2021: APC, -7.5 (-11.4 - -3.6); p = < 0.001) (Table 2).

**Table 2:** Joinpoint analysis of DSH rates per 100,000 person-years by sex and age group during 2000–2021^*^

		IR in 2000	IR in 2021	APC (95% CI)	*P*-value
Males					
	**Age group**				
	10–18 years				
	Segment 2000–2007	54.3	95.3	**8.0 (3.5–12.6)**	0.002
	Segment 2007–2017	95.3	28.2	**-10.6 (-13.6 - -7.6)**	< 0.001
	Segment 2017–2021	28.2	36.9	7.2 (-6.2–22.5)	0.282
	19–24 years				
	Segment 2000–2006	188.4	257.7	**4.2 (1.6–6.9)**	0.005
	Segment 2006–2012	257.7	160.1	**-5.8 (-8.9 - -2.6)**	0.002
	Segment 2012–2015	160.1	87.2	**-18.4 (-31.9 - -2.3)**	0.030
	Segment 2015–2021	87.2	73.9	-2.6 (-6.2–1.0)	0.141
	25–44 years				
	Segment 2000–2013	128.8	107.06	**-2.3 (-3.3 - -1.4)**	< 0.001
	Segment 2013–2016	107.0	64.1	-14.9 (-33.2–8.4)	0.174
	Segment 2016–2021	64.1	50.0	-4.8 (-10.9–1.7)	0.132
	45–64 years				
	Segment 2000–2013	69.1	82.0	0.5 (-0.5–1.5)	0.325
	Segment 2013–2021	82.0	39.3	**-8.2 (-10.5 - -5.9)**	< 0.001
	65 + years				
	Segment 2000–2006	38.5	42.2	2.2 (-2.8–7.4)	0.375
	Segment 2006–2021	42.2	21.7	**-3.4 (-4.6 - -2.2)**	< 0.001
Females					
	**Age group**				
	10–18 years				
	Segment 2000–2009	174.8	331.2	**7.2 (4.2–10.2)**	< 0.001
	Segment 2009–2021	331.2	251.4	**-4.2 (-5.7 - -2.5)**	< 0.001
	19–24 years				
	Segment 2000–2007	259.8	540.9	**11.2 (8.0–14.5)**	< 0.001
	Segment 2007–2012	540.9	509.9	-1.8 (-7.1–3.8)	0.469
	Segment 2012–2016	509.9	225.4	**-18.9 (-26.8 – -10.2)**	0.001
	Segment 2016–2019	225.4	306.0	9.7 (-13.1–38.6)	0.386
	Segment 2019–2021	306.0	257.0	-8.1 (-27.5–16.4)	0.433
	25–44 years				
	Segment 2000–2003	133.6	173.8	**8.9 (1.4–16.9)**	0.023
	Segment 2003–2012	173.8	163.5	-0.7 (-2.2–0.8)	0.337
	Segment 2012–2021	163.5	69.2	**-9.5 (-11.0 - -7.9)**	< 0.001
	45–64 years				
	Segment 2000–2005	87.9	98.9	2.5 (0.0–5.0)	0.051
	Segment 2005–2013	98.9	92.4	-1.3 (-2.7–0.1)	0.065
	Segment 2013–2016	92.4	54.2	**-16.2 (-26.2 - -4.9)**	0.012
	Segment 2016–2019	54.20	48.0	-2.6 (-15.9–12.8)	0.686
	Segment 2019–2021	48.0	35.5	-14.5 (-27.8–1.2)	0.064
	65 + years				
	Segment 2000–2013	42.0	40.4	-0.9 (-2.6–1.0)	0.335
	Segment 2013–2021	40.4	19.6	**-7.5 (-11.4 - -3.6)**	0.001

Abbreviation: IR: Incidence rate; APC: Annual percent change

* Using joinpont analyses, segments with different trends were identified

### DSH methods

Poisoning was the most frequently method for DSH across all age groups and sex (supplementary Fig. 1). Among males, 45–58% of all DSH episodes were by poisoning, while 11–21% and 30–39% were due to cutting and other methods, respectively, when assessing all age groups. Among females, 57–71%, 8–18%, and 11–26% of DSH episodes were due to poisonings, cutting, and other methods, respectively.

**Fig. 3:**
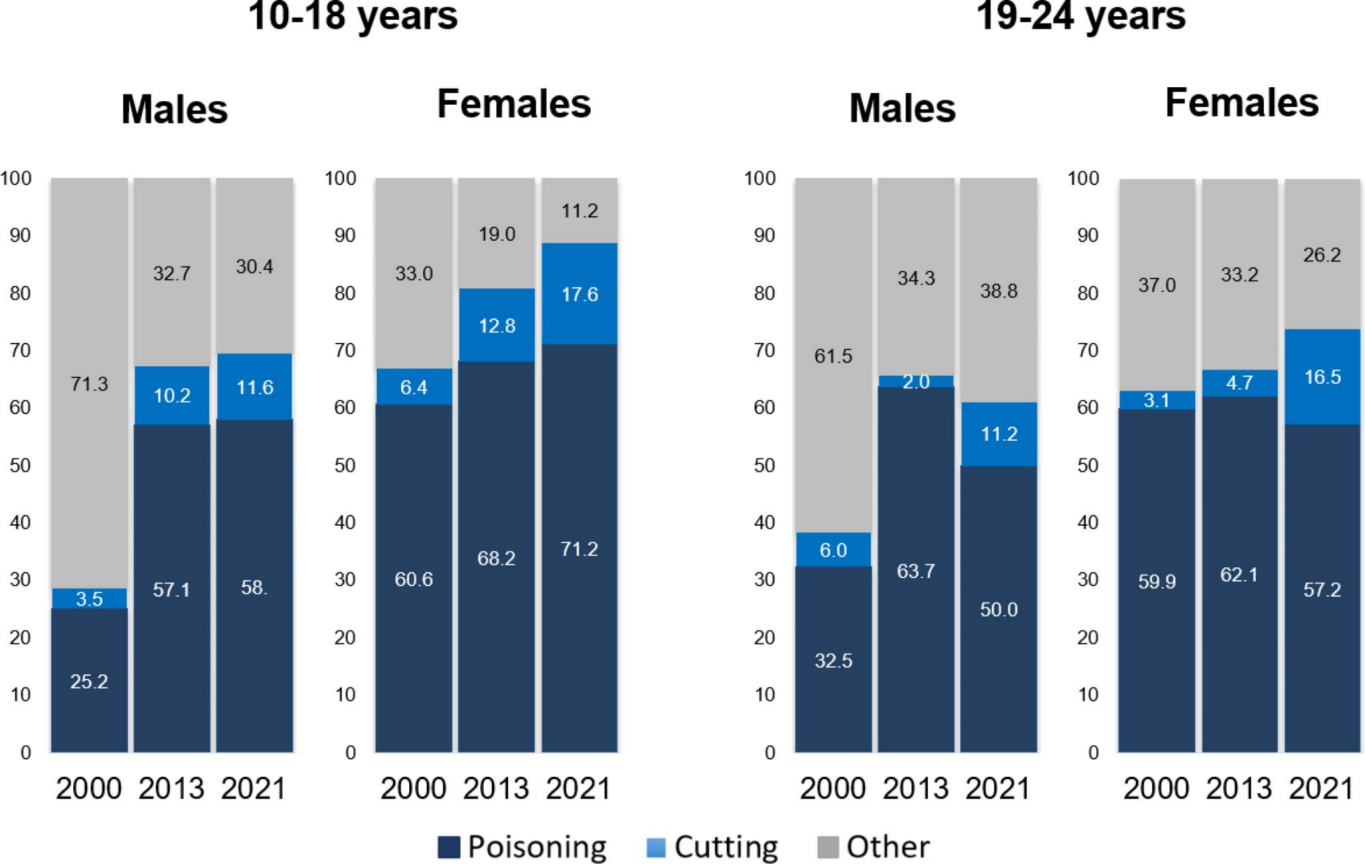
Distribution of DSH suicide method (in percent) for males and females aged 10–18 years and 19–24 years in year 2000, 2013, and 2021

Between 2000 and 2013, the proportion of poisoning episodes increased from 25 to 57% among males aged 10–18 years, whereafter it seemingly stabilized and remained around 58% in year 2021 (Fig. 3). A comparable increase in poisonings from 32 to 64% was observed between 2000 and 2013 for males aged 19–24 years, which declined to 50% in 2021. For females aged 10–18, the proportion of poisonings increased steady from 61% in 2000 to 71%; in 2021. The share of poisonings remained fairly stable with percentages of 60%, 62% and 57% for the years 2000, 2013, and 2021, respectively, among females aged 19–24 years. Additional analyses on distribution of DSH methods by sex and age groups are available in the supplementary material.

## Discussion

In this nationwide, register-based study, the highest rates of DSH were observed for young individuals, in particular females aged 10–18 and 19–24 years. Adolescents and young adults accounted for the major share of DSH episodes, while older adults accounted for the smallest share. In general, rates of DSH were found to decrease during the study period for both sexes and across most age groups. However, fluctuating trends were detected among the youngest age groups of females. The joinpoint analyses revealed between two and five joinpoint segments for different sex and age groups. Significantly decreasing trends with APCs ranging between -18% and − 19% were detected among 19-24-year-olds males and females, respectively. Decreases were also observed for females aged 25–44 years. DSH by poisoning remained the most frequently used method irrespective of sex and age specific differences.

This update on trends of DSH rates in Denmark included one the longest follow-up periods in an international context. The high rates observed for the youngest age groups is supported by previous national and international reports [7, 10, 35]. High rates of DSH have also been demonstrated for teenage girls in Norway [20] and teenagers in Sweden [14]. Opposite, the lowest rates of DSH were been found among older adults, which was also the case in Ireland where female aged 80 years and over had lower DSH rates when compared to those aged 60–69 years [12]. Older adults are generally considered to be more determined and may have planned the act over a longer period of time, which may imply a higher probability of a fatal outcome [36]. In this study, we found a rate ratio of male to female DSH rates for young adults of 2.5, which is higher than the one of 1.7 reported in a recent meta-analysis [19].

The joinpoint analyses revealed increasing APCs for both genders and most age groups until 2006 whereafter overall declining trends were observed. This could rule out a potential adverse effect of the financial recession experienced in Denmark after 2007/8 [11]. It is possible that recessions only had marginal effects on suicidal outcomes in countries with extensive social welfare systems [9]. Further, the findings from this analyses did not suggest that the recent COVID-19 affected rates negatively although more recent data would be needed to determine this with certainty [37]. The decreasing IRs among young adults in Denmark confirmed previous observations [35] and may partially be due to preventive efforts, i.e. an age restriction on OTC non-opioid analgesics effectuated in 2011 and a pack size restriction of the same agents implemented in pharmacies in 2013 [18]. Pack size restrictions of OTC medications have previously been linked to reductions in DSH in several European countries [17, 22].

Proportionally, poisoning was found to be the most frequently used method, which coincides with findings from other countries [20, 22]. The proportion of DSH episodes due to poisonings increased among adolescents over the examined period. Moreover, the proportional increase in cutting among some young age groups could suggest a shift of DSH methods. As new types of drugs may be introduced [38], it is important to monitor the exact drug types, which are being used in DSH events. Unfortunately, this was not possible and underscores the need for more detailed data.

### Implications

The high DSH rates emphasize that preventive efforts to secure further reductions are essential. Effective measures exist, for instance, means restrictions where access to specific types of medication used for poisonings is restrained [18]. This approach can be directed towards specific age groups, such as young individuals, who have high rates and whose DSH episodes predominantly were due to poisoning. Yet, future measures of this type hinge upon accurate identification of the specific drugs used for DSH. Other effort may include access to helplines, safety planning, and psychosocial therapy [39, 40]. Considering the high rate of repetition [4], such supportive tools might be offered to individuals who present to hospital with a DSH episode.

Overall, monitoring of DSH episodes is essential for facilitating preventive efforts [41].

### Limitations and strengths

Strengths of the study included longitudinal, complete, and recent nationwide register data as well as a 21-years follow-up period, which ensured representative and updated findings. Diagnostic codes were uniformly assigned by medical doctors and based on international nomenclature, which increased the external validity. Using a data-driven approach, opposed to visual inspection, improved the reliability of the identified trends.

Limitations should be acknowledged. Hospital registers are compiled for administrative purposes, which may impact data quality in a research context. Records of DSH have been evaluated to under-estimate the true number of self-harm with as much as 30% [7, 42]. Nevertheless, it cannot be excluded that some events might have been recorded twice, e.g. during repeat out-patient presentations. However, there were relatively few of these records. For this reason, the number of joinpoints may have been biased in a conservative direction. Previous evaluations of the algorithm for identifying DSH support its validity albeit it may under-estimate the true figure of DSH [42, 43]. The presented rates may, thus, be viewed as conservative. Individuals who refrained from seeking hospital care after DSH [44] were not included in this study, thus, impacting the external validity of our absolute measures. Assuming that most self-inflicted events in children were accidents [45], we did not include data on children below the age of ten. Detailed information on types of medication, for instance OTC agents, such as non-opioid analgesics, used for overdoses would have been preferred but were not available.

## Conclusion

Overall trends of decreasing DSH rates were identified among Danish males and females during recent years. Nevertheless, the highest rates of DSH were observed among the youngest age groups, particularly for females. The relatively high risk of repetition and fatal outcomes underscore the importance of monitoring and follow-up of individuals who present at hospital with DSH. Despite a potential under-recording of DSH episodes, the proportional increase of poisoning incidents observed among young individuals stresses the need for surveillance and registration of specific methods for DSH in order to inform interventive efforts.

## Electronic supplementary material

Below is the link to the electronic supplementary material.

Supplementary Material 1

## Data Availability

No datasets were generated or analysed during the current study.
