# Supplementary material for: Joinpoint analyses of rates on hospital-recorded deliberate self-harm: an update on Danish national trends
Source: Soc Psychiatry Psychiatr Epidemiol. 2024 Nov 11;60(3):727–36. doi: 10.1007/s00127-024-02795-y (PMC11870884; doi:10.1007/s00127-024-02795-y)
Supplement: Supplementary file 1 — Supplementary Material 1 [file 127_2024_2795_MOESM1_ESM.pdf]

**Joinpoint analyses of rates on hospital-recorded deliberate self-harm:
an update on Danish national trends**

Social Psychiatry and Psychiatric Epidemiology

Britt Reuter Morthorst[1,5,6], *Michella Heinrichsen*[5] & *Annette Erlangsen,*[1,2,3,4]

[1] Danish Research Institute for Suicide Prevention, Mental Health Centre Copenhagen, Copenhagen, Denmark
[2] Department of Mental Health, Johns Hopkins Bloomberg School of Public Health, Baltimore, MD, USA
[3] Copenhagen Research Centre for Mental Health, Copenhagen, Denmark
[4] Center of Mental Health Research, Australian National University, Australia
[5] Research Unit, Child and Adolescent Mental Health Center, Copenhagen University Hospital – Mental Health Services CPH, Copenhagen, Denmark
[6] Department of Clinical Medicine, Faculty of Health, University of Copenhagen, Denmark

Corresponding author: Britt.reuter.morthorst@regionh.dk

Supplementary figures. Distribution of DSH suicide method by sex and age group (in percent) for the years 2000, 2013, 2017, and 2021.

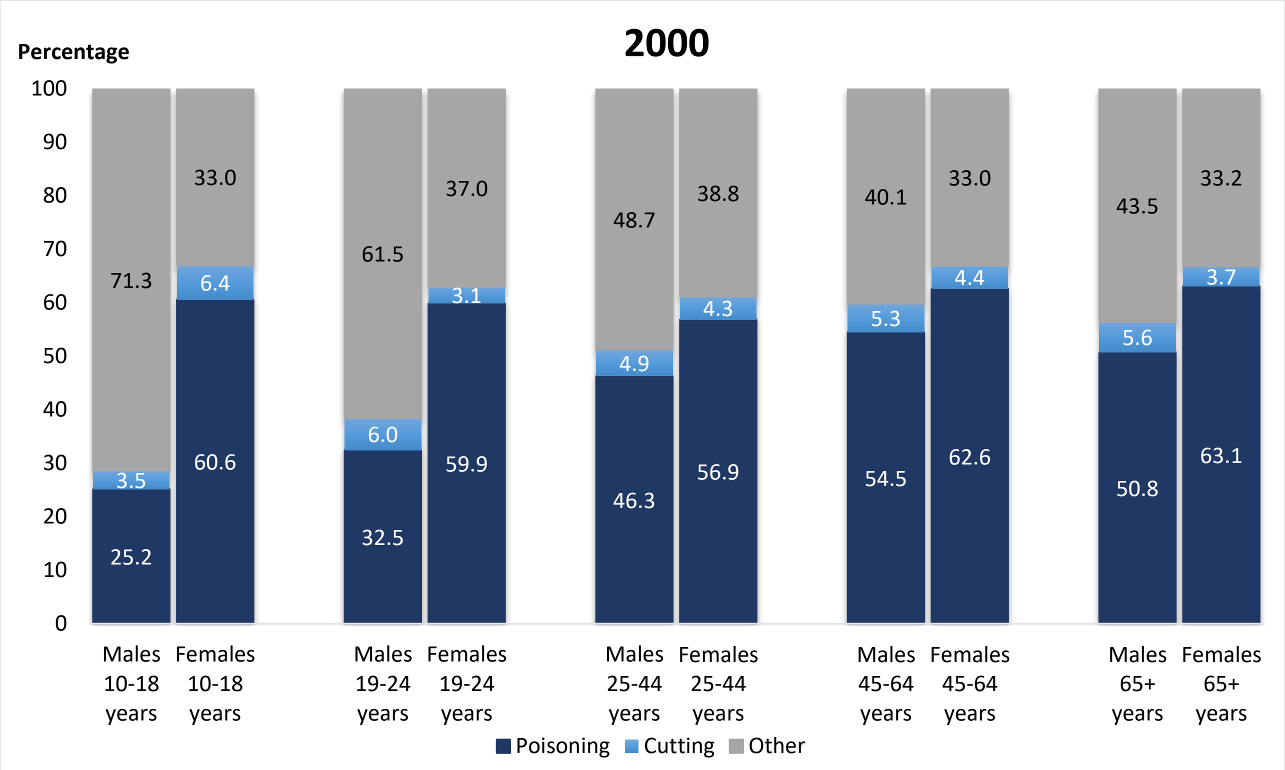

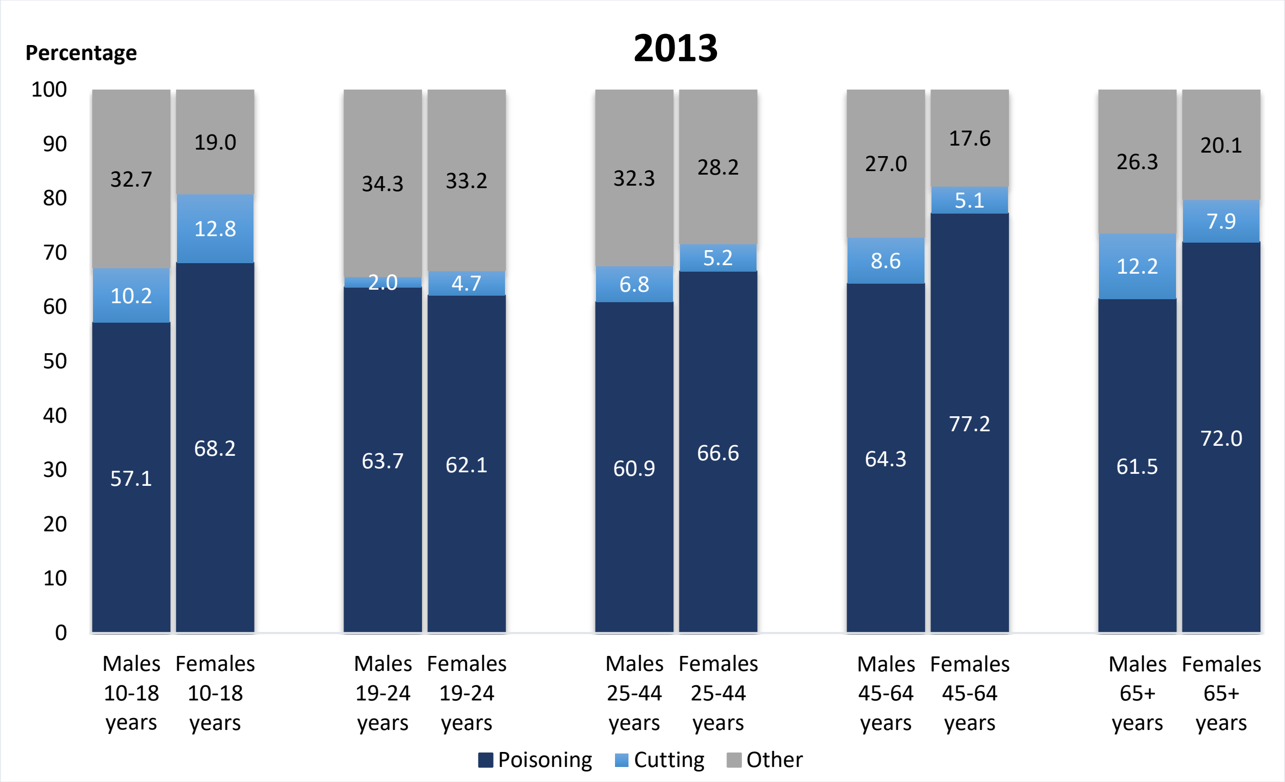

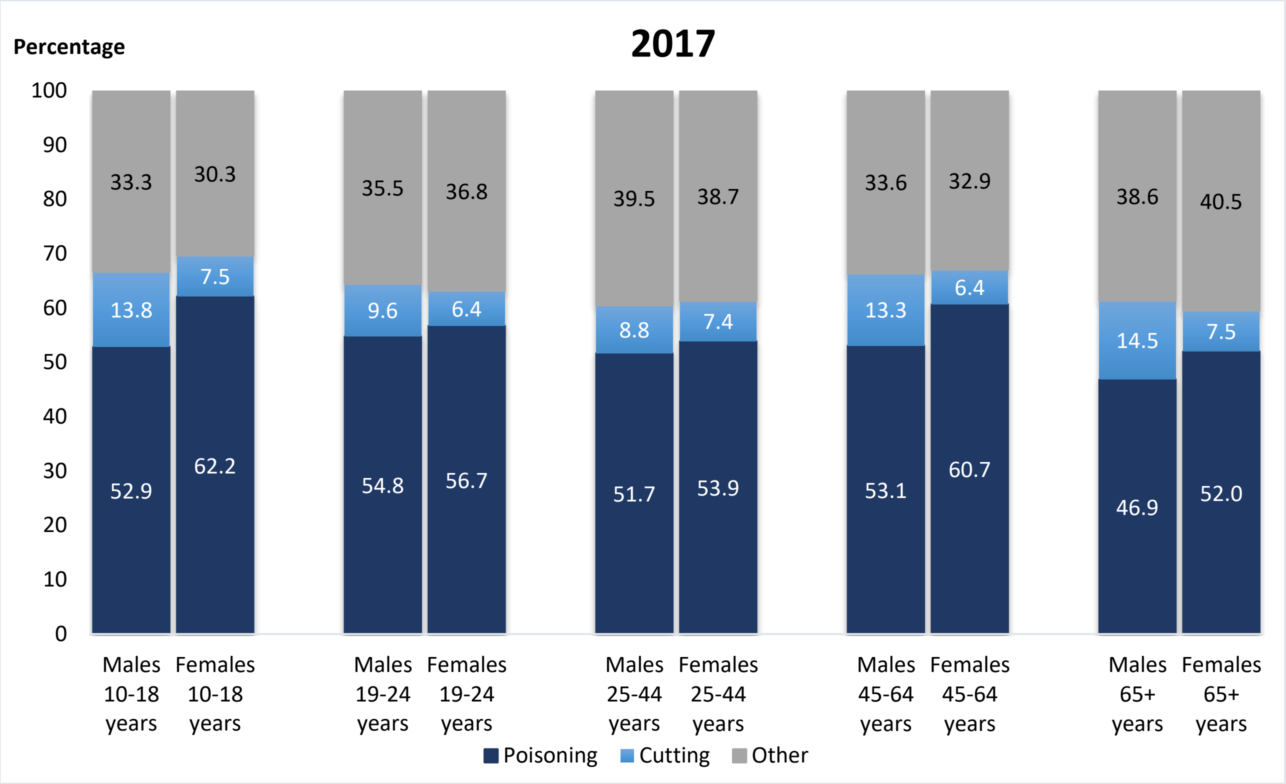
2017
Percentage
100
90
80
70
60
50
40
30
20
10
0
33.3
13.8
52.9
30.3
7.5
62.2
35.5
9.6
54.8
36.8
6.4
56.7
39.5
8.8
51.7
38.7
7.4
53.9
33.6
13.3
53.1
32.9
6.4
60.7
38.6
14.5
46.9
40.5
7.5
52.0
Males 10-18 years
Females 10-18 years
Males 19-24 years
Females 19-24 years
Males 25-44 years
Females 25-44 years
Males 45-64 years
Females 45-64 years
Males 65+ years
Females 65+ years
Poisoning
Cutting
Other

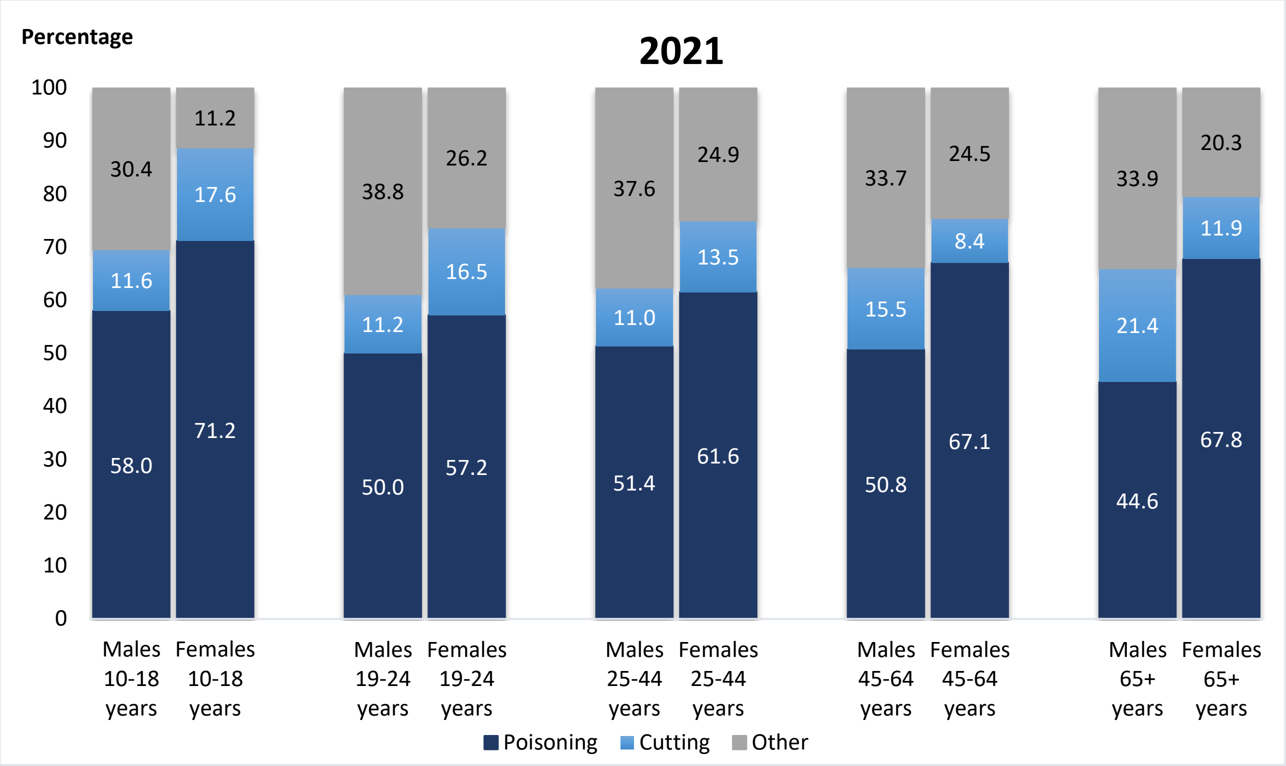
2021
Percentage
100
90
80
70
60
50
40
30
20
10
0
30.4
11.6
58.0
11.2
17.6
71.2
38.8
11.2
50.0
26.2
16.5
57.2
37.6
11.0
51.4
24.9
13.5
61.6
33.7
15.5
50.8
24.5
8.4
67.1
33.9
21.4
44.6
20.3
11.9
67.8
Males 10-18 years
Females 10-18 years
Males 19-24 years
Females 19-24 years
Males 25-44 years
Females 25-44 years
Males 45-64 years
Females 45-64 years
Males 65+ years
Females 65+ years
Poisoning
Cutting
Other
